# KLINSE: a comprehensive service model for rare disease information and care management support

**DOI:** 10.1186/s13023-026-04217-5

**Published:** 2026-01-31

**Authors:** Katrin Jäger, Elke Dannenmann-Stern, Sevda Inbasi, Ute Grasshoff, Christina Vossler-Wolf, Holm Graessner

**Affiliations:** University Hospital Tübingen, Institute for Medical Genetics and Applied Genomics and Center for Rare Diseases, Calwerstr. 7, 72076 Tübingen, Germany

**Keywords:** Rare diseases, Care management support, Diagnosis-informed therapy, Clinical Information Center for Rare Diseases

## Abstract

**Background:**

Researching rare disease (RD) knowledge is often labor-intensive and requires familiarity with a wide array of national and international databases, making it impractical in routine clinical settings and frequently insufficient. To address the gap between diagnosis and the urgent need for information on diagnosis-informed therapy and care management, the Clinical Information Center for Rare Diseases (KLINSE) was established in 2021 at the Center for Rare Diseases Tübingen. Designed as a clinician-to-clinician service, KLINSE provides up-to-date clinical knowledge and management recommendations for RDs in a timely and structured manner.

**Results:**

Between its start of operation in May 2021 and November 2023, KLINSE received 100 case submissions, of which 88 were accepted for processing. The majority involved ultra-rare diseases (prevalence < 1:1,000,000) and predominantly affected children and adolescents. In 58% of cases, KLINSE was contacted within one year of genetic diagnosis, while delays of up to 10 years were noted in others. KLINSE supplied information specifically requested by referring clinicians and additionally provided unsolicited yet clinically relevant insights. The most common inquiries related to treatment options, clinical trials and registries, centers of expertise, and patient organizations. Standardized feedback highlighted KLINSE’s high utility and value to clinical practice.

**Conclusion:**

Our findings underscore persistent deficits in accessible disease-specific information for rare conditions. The positive reception of KLINSE demonstrates the critical role of low-threshold, expert-driven services in enhancing patient care and easing the burden on clinicians managing complex RD cases.

## Background

### Rare diseases

In the European Union, disorders affecting fewer than 5 in 10,000 individuals are classified as rare diseases (RD) [1]. However, the majority of diagnosed RDs involve conditions with even fewer patients; approximately 85% of RDs affect fewer than 1 in 1,000,000 people [2]. The total number of rare diseases is estimated to exceed 8,000, with approximately 250 new diseases identified each year [3]. Consequently, the aggregate number of individuals affected by RDs is substantial. In Germany alone, an estimated four million people are living with a rare disease (patients with rare diseases, PwRD) [4].

Despite their collective prevalence, individual PwRDs often face unique challenges related to their condition. On average, it approximately takes between 5 and 8 years − 4.5 years for children (≤ 18 years) and 8.2 years for adults in Germany [5] - to obtain an accurate diagnosis [2]. Genetic testing plays an increasingly vital role in the diagnostic process, as 70% of RDs have a genetic etiology. The advent of high-throughput next-generation sequencing (NGS) technologies in diagnostic laboratories has markedly improved the diagnostic landscape for RDs in recent years.

### Need for adequate diagnosis-Informed therapy and care management

Achieving a diagnosis is the first step towards providing appropriate disease-specific care. However, the subsequent steps - diagnosis-informed therapy and care management - are frequently inadequate. A significant barrier is the lack of accessible, up-to-date knowledge and information. Physicians operating outside Centers of Expertise (CoE) often lack the experience and expertise needed to treat specific RDs. Furthermore, clinical practice guidelines or treatment recommendations from professional organizations are often unavailable. This deficit is exacerbated by several factors, including the vast number of RDs, the multitude of potential genetic causes, the heterogeneity of disease manifestations, genotype-phenotype correlations, and the rapid evolution of knowledge in this field.

As a result, many PwRD face a “management and treatment odyssey” following the resolution of the diagnostic odyssey. Physicians seeking information on management and treatment options for RDs often must undertake extensive and labor-intensive research, requiring familiarity with various national and international databases and resources. In many cases, it is necessary to directly identify and consult an expert or CoE.

This research burden is untenable in routine clinical practice. For instance, German general practitioners, who see an average of 52 patients per day with a mean consultation time of 7.6 min per patient [6, 7], and specialist practitioners, who see an average of 38 patients daily [6], lack the time to conduct such detailed investigations.

As a result, despite advancements in the diagnosis of RDs, subsequent disease-specific treatment and care management are often absent, suboptimal, or delayed.

### Clinical Information Center for Rare Diseases (KLINSE)

The Clinical Information Center for Rare Diseases (KLINSE) was established in 2021 as a service of the Center for Rare Diseases Tübingen to address this critical unmet need. KLINSE provides a low-barrier expert knowledge resource for physicians treating patients with genetic RDs. Structured as a clinician-to-clinician service, KLINSE delivers the most current information on RD clinical knowledge, treatment and management in a timely manner.

This study presents an analysis of the first 100 patients for whom KLINSE was consulted, offering insights into the service’s impact on addressing the challenges associated with RD treatment and care management.

## Methods

### Targeted outreach and engagement

Since approximately 70% of rare diseases (RD) have a genetic basis, and KLINSE operates as part of the Center for Rare Diseases (Zentrum für Seltene Erkrankungen, ZSE) Tübingen of which the Institute of Medical Genetics and Applied Genomics is a member institution, our initial outreach focused on internal genetic services. Flyers were distributed alongside genetic testing reports to referring physicians and clinics. However, this approach yielded minimal response.

Recognizing that many genetically driven rare diseases manifest in childhood or adolescence, we redirected our efforts to healthcare facilities specializing in pediatric care. Social pediatric centers across Baden-Württemberg were informed about KLINSE’s services through written communications and personal meetings. Subsequently, selected ZSEs, clinics with neuropediatrics departments, and medical centers for adults with disabilities (MZEBs) were also contacted.

Additionally, KLINSE was introduced in further education training sessions conducted by the Academy for Rare Diseases Education that belongs to ZSE as well as during the German National Conference for Rare Diseases in 2021. These initiatives aimed to spread awareness for KLINSE among medical professionals and reached participants from pediatrics, genetics, neurology, ophthalmology, and other specialties.

### KLINSE process

**Fig. 1:**
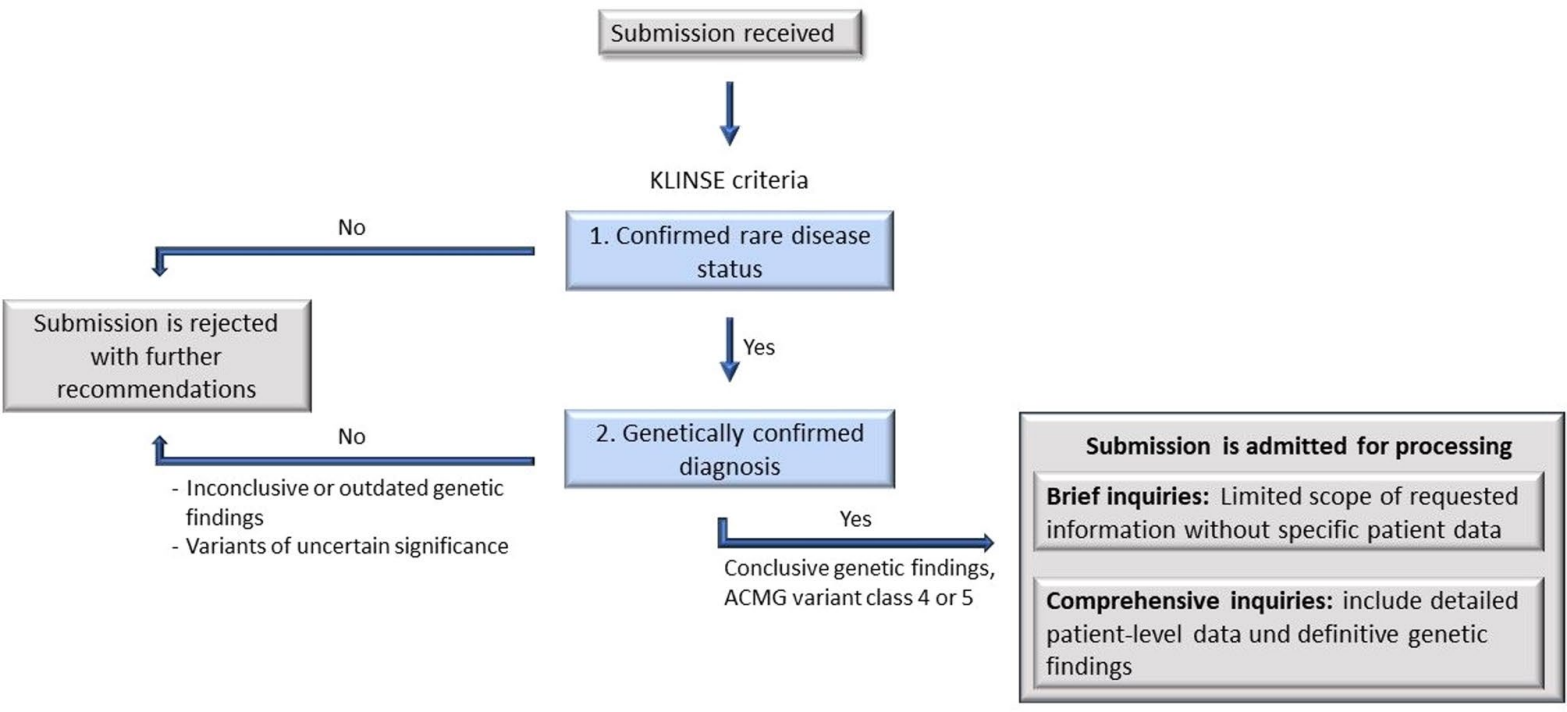
KLINSE Process. ACMG: American College of Medical Genetics and Genomics

Upon receiving a submission, inquiries are systematically reviewed for processing eligibility (see Fig. 1). The criteria for acceptance include (1) confirmed rare disease status and (2) a verified genetic basis for the disease. To assess these criteria, genetic testing reports and signed data privacy consent forms (from both the treating physician and the patient or legal representative) are required. Additional documentation, such as recent medical reports and genetic counseling notes, is also requested to facilitate in-depth evaluation.

Submissions failing to meet these criteria are rejected with written explanations, and submitters may be referred to an appropriate center. Accepted submissions are assigned a case number, recorded in our database for statistical purposes, and categorized as follows:

- **Comprehensive Inquiries**: Include detailed patient-level data and definitive genetic findings.
- **Brief Inquiries**: Limited scope without specific patient data.
- **Unresolved Genetic Findings**: Cases involving outdated genetic testing or variants of uncertain significance (VUS).

### Research strategies for RD treatment and care management

To address specific inquiries from referring physicians, KLINSE employs a systematic research process to gather information on various topics:

- Genetics and molecular mechanisms.
- Clinical presentation and genotype-phenotype correlation.
- Treatment and management options.
- Recommended surveillance.
- Natural history and prognosis.
- Guidelines.
- Individual scientific experts and clinical centers of expertise.
- Clinical trials, registries, and research projects.
- Patient support groups and networks.

Research is conducted using a pre-defined KLINSE protocol referring to established databases and resources such as Orphanet, OMIM, PubMed, GeneReviews, and clinical trial registries, among others, as well as disease-specific and cross-disease networks and databases. Furthermore, identified leading experts are being contacted. The quality of information recovered is appraised by KLINSE clinicians.

Information is compiled into a standardized information letter tailored to each case, containing sources and dates for each given information. The letter undergoes a thorough review process before being sent via email (excluding patient-specific data) and postal mail (for comprehensive cases). A feedback form is included to evaluate the usability and quality of the provided information. The information letter is, furthermore, supplemented by pertinent publications if existing and available via open access.

### Follow-up

To ensure the accessibility of the KLINSE service and to minimize the administrative burden placed on referring physicians, no structured follow-up was initially included in the study design. In order to retrospectively evaluate the clinical relevance of KLINSE’s work and to identify possible alterations in patient care pathways, feedback from the center with the highest number of submissions was subsequently requested.

### Data management and resource development

Each accepted case is assigned a unique case number and anonymized patient identifier. Patient-specific and inquiry-specific data are stored separately in secure Excel files accessible only to KLINSE team members. Disease-related information collected during research, including OMIM and Orphacodes, is entered into a custom REDCap database with reference to the according gene.

The goal of this database is to facilitate future collaboration and provide a resource for other research groups and the general public. Any shared data are carefully de-identified to ensure patient privacy. Cases not meeting the criteria for acceptance are not entered into the database but are documented for tracking purposes.

### Updates

When new information relevant to patient care becomes available regarding a specific case, this information is communicated to the referring physician or the patient at a later time. Additionally, referring physicians are encouraged to submit follow-up inquiries if limited information is initially available on the specific condition.

## Results

### KLINSE case submissions (May 2021–November 2023)

From its inception in May 2021 until November 2023 KLINSE received the first 100 case submissions. These were distributed as follows: 20 cases in 2021 (May–December), 34 cases in 2022 (January–December), and 46 cases in 2023 (January–November).

Of these, 88 cases were accepted for processing. Among these, 66 included complete individual patient-level data and confirmed genetic-diagnostic information and were thus processed as comprehensive inquiries, while 22 were processed as brief inquiries without such patient level data (see Fig. 1). 12 submissions were not accepted due to inconclusive genetic findings, variants of uncertain significance, or failure to meet KLINSE criteria. In these instances, submitters were advised to repeat genetic testing, re-evaluate findings within two to three years of initial genetic testing or were referred to an appropriate healthcare provider if KLINSE criteria were not met.

### Descriptive analysis of submitters and cases

Submissions were received from a diverse range of healthcare providers:

- Social pediatric centers (48 cases).
- Human/medical genetics centers (24 cases).
- General specialist practitioners (15 cases).
- Hospitals (7 cases).
- A medical center for adults with disabilities (1 case).
- Directly from patients (5 cases).

**Fig. 2:**
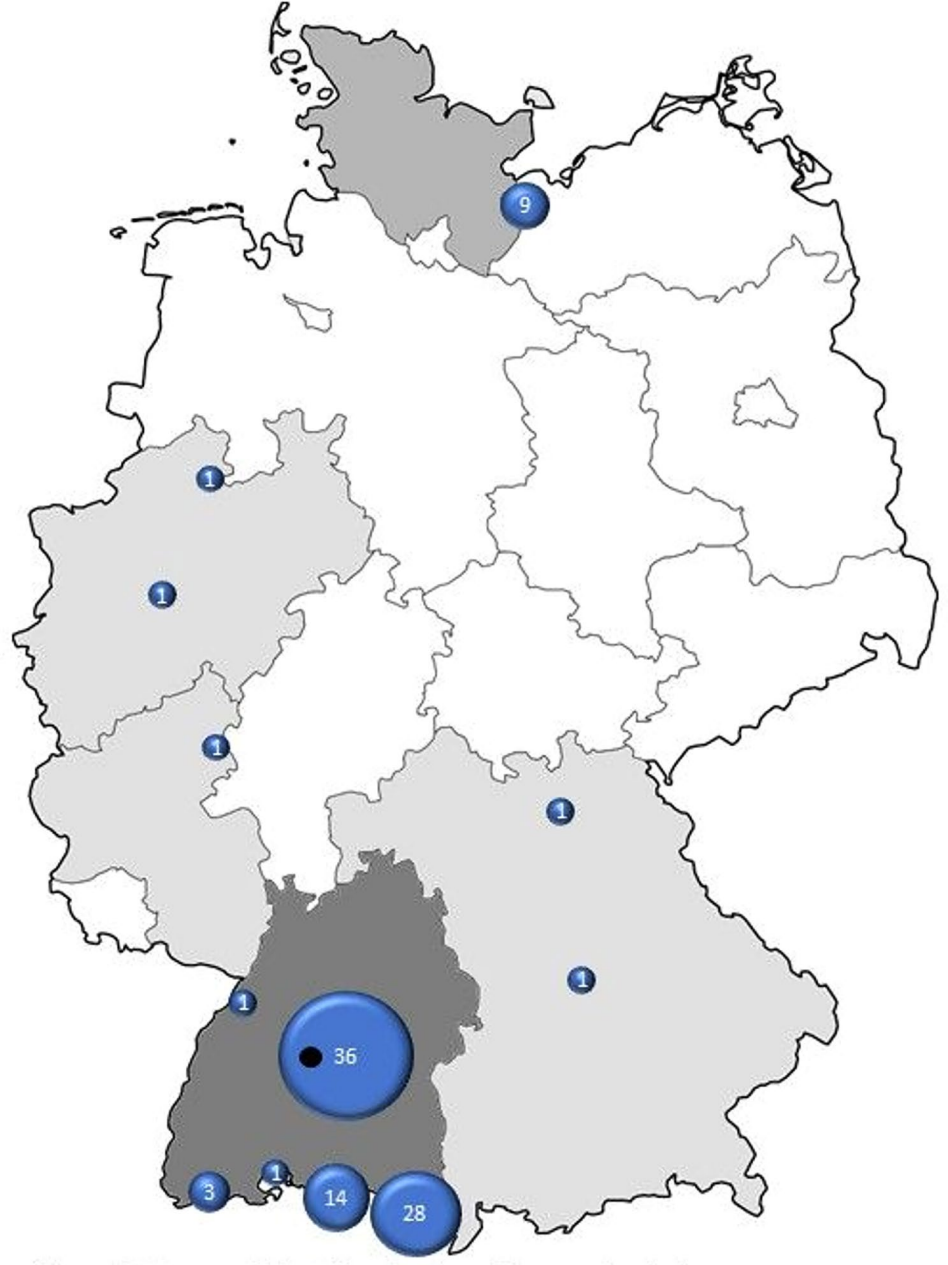
Geographical distribution of case submissions. Location of KLINSE indicated in black

Due to the geographic focus of our outreach efforts, the majority of cases originated in the German federal state of Baden-Württemberg (see Fig. 2).

### Patient demographics and disease characteristics

The majority of submissions (71%) pertained to diseases with a prevalence of < 1:1,000,000. For 46 submissions, fewer than 100 cases had been published in the literature or reported in disease-specific support groups.

**Fig. 3:**
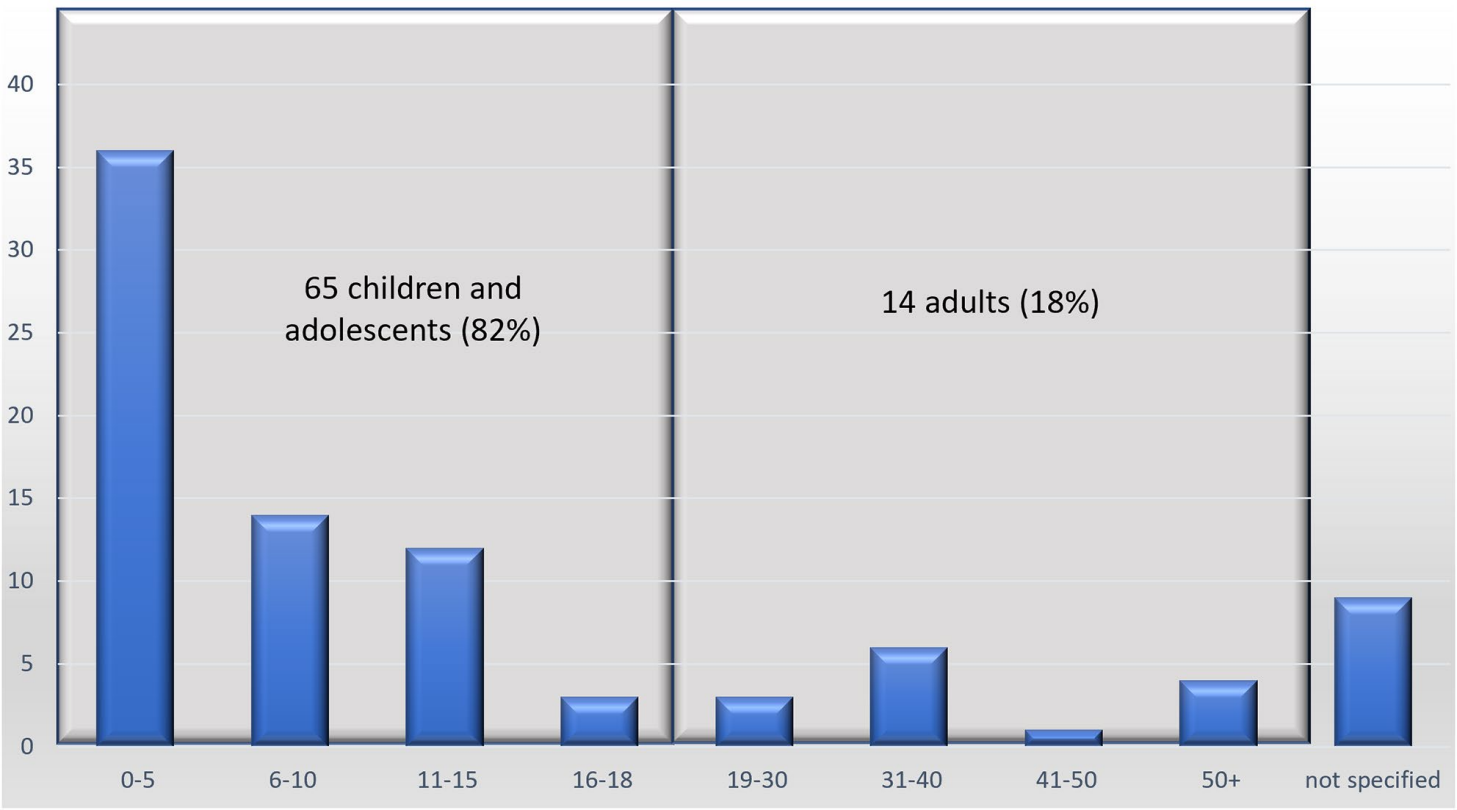
Age distribution

Gender data were available for 93% of patients (82/88): 55% (45/82) were male and 45% (37/82) were female. Age data (see Fig. 3) were available for 90% of patients (79/88), with a mean age of 12.4 years and a median age of 7 years (range: 10 months – 62 years). Of these, 65 cases (82%) involved children and adolescents (0–18 years).

### Molecular genetic findings and phenotypes

A complete genetical work-up was documented in 66 cases: 53 pathogenic or likely pathogenic variants in single genes, 10 chromosomal disorders, 2 pathogenic variants in the mitochondrial genome, and 1 case included a pathogenic variant in a single gene as well as a pathogenic variant in the mitochondrial genome. 22 cases were processed without patient-level genetic information.

The predominant phenotypic categories and their associated genes/chromosomal aberrations are summarized in Table 1. Disease groups and according genes/CNV aberrations:

**Table 1:** Disease groups and according genes/CNV aberrations (for classification purposes, we decided on the predominant phenotype or the main feature of the disease)

Disease groups	No	Genes and chromosomal aberrations, syndromes
Neurodevelopmental disorders	38	Single gene disorders: AGO2, AHDC1, ASXL3, AUTS2, CHD3, CLTC, EP300, FBXO11 (2x), FOXG1, GRIN2B, KAT6A, KDM6B, MED13L, MTOR, NAA-10 (2x), NR4A2, NSD2, PHF8, POU3F3, PPP3CA, RHOBTB2, RORA, SETBP1, TBL1XR1 CNV disorders: Deletion 1p21, Microdeletion 1p36.33p36.32, Microdeletion 1q44, Microdeletion 2q31.3q32.1, Microdeletion 2q33.1, Microdeletion 3p26.1p25.3, Deletion 9p24.3p23, Microdeletion 15q11.2q12, Microdeletion 15q25.1q25.2, Microdeletion 16p11.2, Microdeletion 22q11, Microduplication Xq28
Neurologic disorder	15	ATM (2x), ATP1A3, CHD2, DEPDC5, GJB1, KCNA1, KCNB1, KMT2B, POU4F1, RARB, SCN9A, SORD1, TSEN54, WDR45
Metabolic disorders	10	ABCC8, AHCY, CDC73, CYP24A1 (2x), HGD, HPRT1, LAMP2, NKX2-1, PHKA2
Skin, connective tissue or vascular diseases	9	ABCC9, ACAN, AKT1 (2x), ATP2A2, FBN1, PTEN, SMAD4, Klippel-Trenaunay syndrome
Ophthalmologic diseases	4	ABCA4, NDP, OPA1 (2x)
Muscular diseases	4	LMNA (2x), RyR1, SELENON (SEPN1, SELN)
Other diseases	8	CYB5R3, ERCC8, KIF11, MT-TL1 (2x), MYCN, PDE4D, STK11

Number of inquiries is indicated by (2x) if more than one. CNV: copy number variation

- **Neurodevelopmental disorders** (38 cases).
- **Neurologic diseases** (15 cases).
- **Metabolic disorders** (10 cases).
- **Skin**,** connective tissue**,** and vascular diseases** (9 cases).
- **Ophthalmologic diseases** (4 cases).
- **Muscular diseases** (4 cases).
- **Other diseases** (8 cases).

### Time between genetic testing and KLINSE contact

Data were available for 66 cases. The time between date of genetic diagnosis as specified in the genetic report and first KLINSE contact was analysed. In 58% (38/66) of cases, KLINSE information was requested within one year of genetic testing. Delays of 1–7 years were documented for the remainder, with two cases reporting delays of 10 years.

### Core and specific services provided

As a core service, KLINSE provides information regarding general disease information for each submitted case, specifically:

1. Clinical presentation of the disease.
2. Molecular disease mechanisms.
3. Genotype-phenotype correlation if applicable.

KLINSE’s specific services include comprehensive data tailored to the following areas and as requested by the submitting clinician, if available through the KLINSE research:

1. Treatment and management options.
2. Recommended surveillance.
3. Natural history and prognosis.
4. Guidelines.
5. Individual scientific experts and clinical centers of expertise.
6. Clinical trials, registries, and research projects.
7. Patient support groups and networks.

**Fig. 4:**
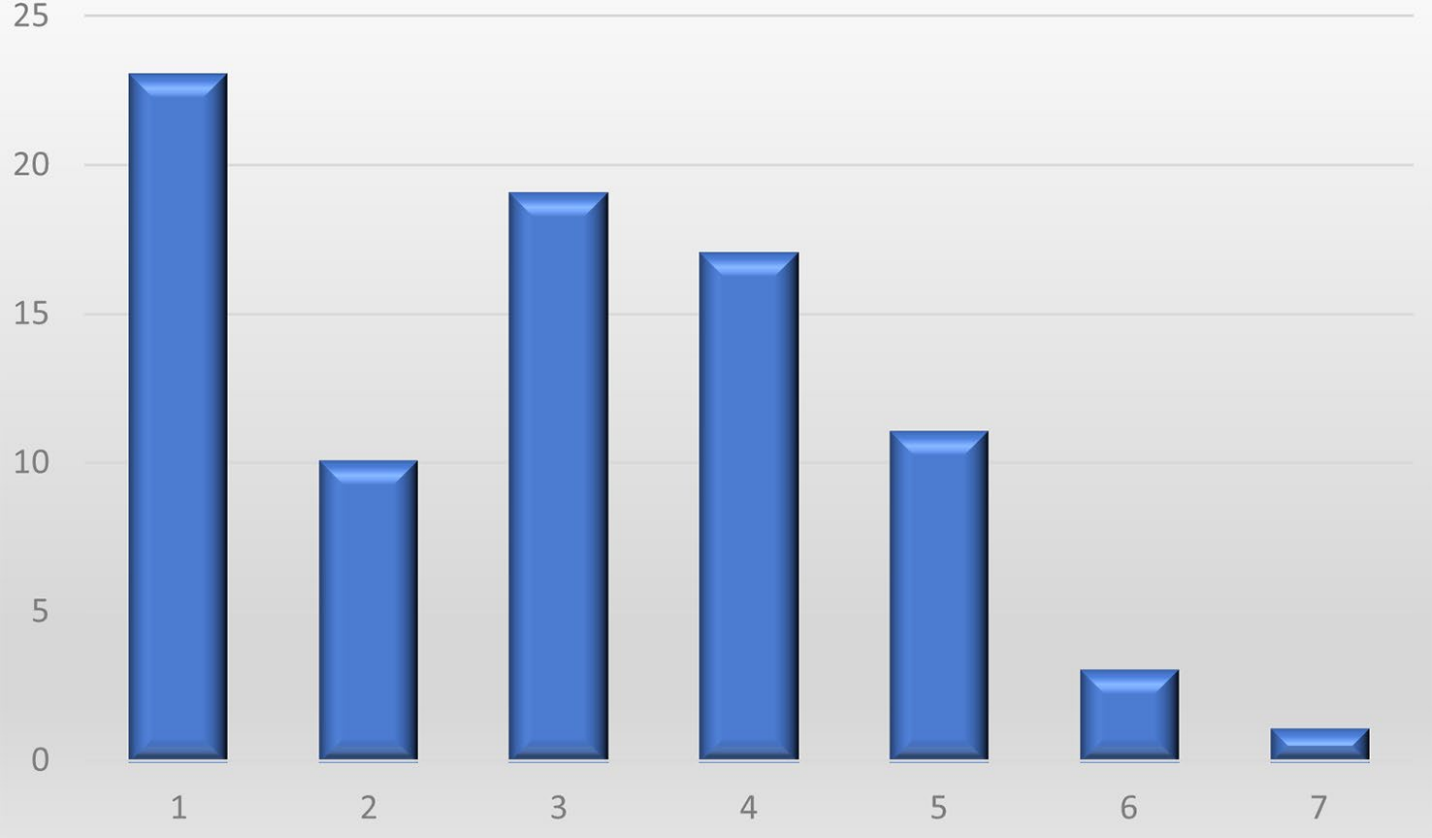
Number of requested information on KLINSE’s core and specific services

The most frequently requested information pertained to treatment options (37/88), clinical trials and registries (36/88), clinical centers of expertise (35/88) as well as patient groups and networks (32/88). While 26% of submitting clinicians (23/88) requested information regarding just one of KLINSE’s specific services, 74% of submitters (65/88) indicated two or more specific services (see Fig. 4).

**Fig. 5:**
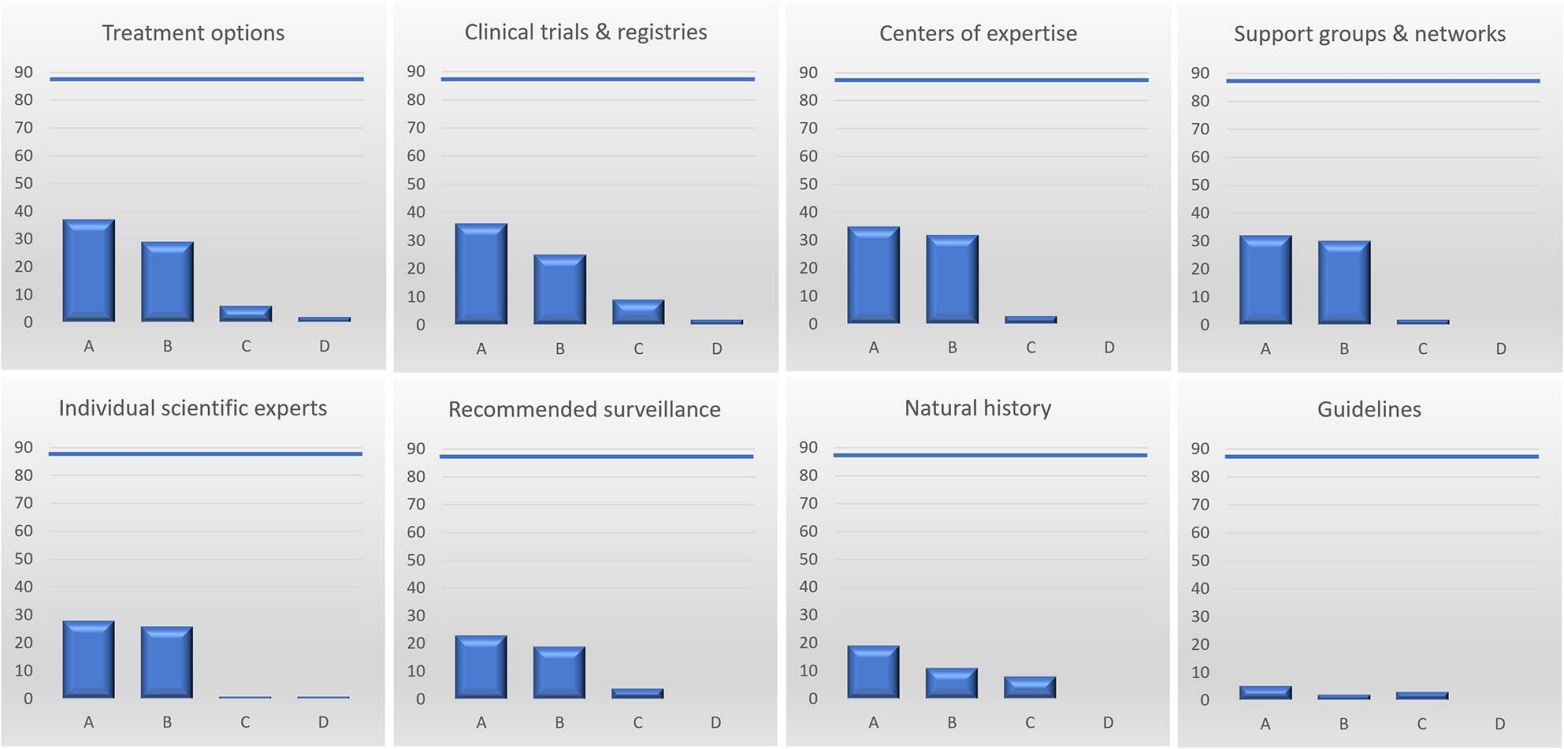
Availability of requested information. (**A**) How often was the information requested. (**B**) How often was the requested information available. (**C**) How often was the requested information not available. (**D**) Submitters were referred to a center of expertise for specific information. Legend: blue bar represents the total number of processed inquiries (88)

While core information regarding clinical characteristics and genetical information was available for all cases, information and data regarding specific knowledge areas were found much less frequently (see Fig. 5). For example, information on natural history was found in only 58% of cases, reflecting the prevalence of ultra-rare diseases among submissions. Information on treatment options was available in 78% of cases, including general and specific therapeutic recommendations. Specific information on mechanistic and/or disease modifying therapies was available in 4 cases.

In addition to specific information that was asked for, KLINSE also provided information that was considered particularly important for patient care even if it was not specifically requested. These specific services included information on treatment options in 24 cases, clinical centers of expertise in 20 cases, individual scientific experts in 27 cases and patient support groups and networks in 41 cases. Altogether, unsolicited information was provided for 50 submissions, corresponding to 57% of all submissions.

### Feedback and evaluation

Standardized feedback was received for 40 of 88 cases.

- **Question resolution**: 95% (38/40) of submitters indicated all questions were fully answered; 5% (2/40) indicated partial resolution.
- **Scope of information**: 97.5% (39/40) of respondents found the scope exceeded or met expectations; 2.5% (1/40) found it partially sufficient.
- **Practical relevance**: 77.5% (31/40) found the information relevant for patient care, 17.5% (7/40) partially relevant, and one case unclear.

Categories deemed most helpful included CoEs and experts, existing networks and support groups, and general disease information.

### Follow-Up

Feedback regarding clinical relevance of KLINSE’s work and alterations in patient care pathways was requested from the social pediatric center with the highest number of overall submissions (24 submissions). They find high relevance in KLINSE’s work as KLINSE research generates insights and outcomes that exceed the institutional scope and methodological capacities of a non-university healthcare provider. Specifically regarding following aspects:

- Professional literature research and analysis addressing specific clinical questions.
- Direct contact with international experts specialized in the condition.
- Collaboration and communication with global research and study groups.
- Evaluation of available therapeutic options and guidance on access to these treatments.
- Networking with international patient organizations.
- Provision of key recommendations for additional diagnostic assessments and preventive measures.

3 cases were specifically mentioned as indicated in Table 2. Alterations in patient care pathways after KLINSE contact.

**Table 2:** Alterations in patient care pathways after KLINSE contact

Gene	Rechercheergebnis der KLINSE	Alteration in patient care pathways
**TBL1XR1**	Possible cerebral involvement with this genetic defect, MRI of the brain recommended	Initiation of an MRI of the brain with the following clinically relevant findings: Hydrocephalus due to Chiari malformation. Follow-up recommended after 2 years, or earlier if new symptoms occur
**RHOBTB2**	Correct diagnosis: RHOBTB2-associated DEE with severe therapy-refractory epilepsy, DEE-64	Specification and differentiation of the correct diagnosis
**ATP1A3**	Identification of a specialized expert center and establishment of contact with it	Contact with the expert center as a treating institution for children and adults and initiation of co-management there, transition to adult medicine

MRI: magnetic resonance imaging; DEE: developmental and epileptic encephalopathy

## Discussion

This analysis of the first 100 case submissions to KLINSE underscores the pressing need for clinician-to-clinician services that address gaps in knowledge and support for the management of RD. To our knowledge, we were the first to establish this kind of service. Despite advancements in genetic testing, our findings highlight persistent challenges in post-diagnostic care, particularly for ultra-rare conditions with limited published data and available information. Compared to diagnostic genetic reports and genetic counselling that focus on communicating and explaining the diagnosis (to patients and families), the post-diagnostic data and information provided by KLINSE are on clinician level and both much broader in scope and tailored to the specific inquiry.

The demographic characteristics of patients served by KLINSE align with the broader trends observed in RD research, where pediatric populations represent a significant proportion of newly diagnosed affected individuals [8]. However, based on the limited experience gained through KLINSE, the service can also be applied to the adult population. The predominance of neurodevelopmental disorders among cases is consistent with the high genetic contribution to this category, emphasizing the importance of genetic insights in guiding clinical management [9, 10].

### Challenges in knowledge dissemination

Our findings reveal critical deficits in the availability of disease-specific information, particularly concerning information on CoEs and experts, general disease information and even disease-specific networks and support.

While core information was consistently available, specific data - such as natural history of the disease and therapeutic recommendations - were often lacking for ultra-rare diseases. This underscores the need for robust international collaboration and knowledge-sharing platforms to enhance data accessibility for clinicians worldwide.

The extended delays between genetic diagnosis and contact with KLINSE in some cases highlight another significant challenge: the gap between diagnostic advancements and their integration into clinical care that reflects the current state of knowledge [11, 12]. These delays may reflect limited awareness of available resources like KLINSE and RDs in general, a lack of time in clinical practice for thorough case reviews, or the inherently slow pace of translating genetic findings into actionable care strategies.

### Utility of KLINSE services

Feedback from submitters demonstrates the high utility of KLINSE in addressing these challenges. Nearly all respondents reported that the information provided met or exceeded their expectations and was relevant to patient care. These results validate the KLINSE model as an effective approach to bridging the gap between genetic diagnosis and actionable treatment and management strategies.

The success of KLINSE also emphasizes the value of low-barrier access to expert knowledge. The service’s comprehensive approach, combining general disease information with tailored, specific guidance, addresses both immediate and long-term needs of physicians managing RD patients.

### Limitations and future directions

This study has several limitations. First, the geographic focus on Baden-Württemberg limits the generalizability of findings to other regions with differing healthcare infrastructures. Second, the relatively small sample size does not capture the full spectrum of RD cases encountered in clinical practice.

Additionally, to date, no structured patient-centered follow-up has been established, thus it is currently not possible to assess the whole extent to which the activities of KLINSE have modified and expedited patient pathways. To address this point, KLINSE’s feedback form has been updated to include an additional item asking the referring physician to describe any alterations in patient care pathways resulting from KLINSE’s input.

The majority of inquiries related to the pediatric population. However, since approximately half of all individuals affected by rare diseases are adults, a need for information can also be assumed in this group. KLINSE has also attempted to identify potential needs in this area through contact with MZEBs. However, the response has so far been rather limited, which may be due to the fact that genetic diagnostics are not yet as widely established in the adult sector.

Future efforts should aim to expand KLINSE’s reach, both geographically and across diverse healthcare settings. Additionally, increased collaboration with international RD networks and infrastructures such as Treatabolome [13, 14] resources and the integration of artificial intelligence tools for data mining could enhance the breadth and efficiency of information retrieval. In the long term, KLINSE aims to secure institutional health system funding to ensure the model’s sustainability and enable broader scalability within routine clinical practice. Building on the current results, KLINSE will be able to engage in discussions with health insurance providers, as the demonstrated effectiveness and feasibility may serve as a foundation for potential reimbursement models and formal integration into health care services.

## Conclusion

The establishment of KLINSE has provided a valuable resource for healthcare providers managing patients with rare diseases. Our analysis demonstrates the service’s ability to fill critical gaps in knowledge dissemination and support, particularly for ultra-rare conditions.

By offering timely, expert-driven insights, KLINSE not only facilitates better patient outcomes but also reduces the burden on individual clinicians navigating the complex landscape of RD care. Continued investment in such initiatives, coupled with efforts to improve data availability and foster global collaboration, is essential for addressing the unique challenges posed by rare diseases.

These findings underscore the importance of scalable, clinician-focused support systems that bridge the gap between diagnostic advancements and clinical care, ultimately contributing to improved quality of life for patients with rare diseases.

## Electronic Supplementary Material

Below is the link to the electronic supplementary material.

## Data Availability

Not applicable.

## Abbreviations

CoE: Center of Expertise
KLINSE: Klinische Informationsstelle für Seltene Erkrankungen, Clinical Information Center for Rare Diseases
MZEB: Medizinische Zentrum für Erwachsene mit Behinderung, Medical Center for Adults with Disabilities
NGS: Next-Generation Sequencing
PwRD: Patients with Rare Diseases
RD: Rare Diseases
VUS: Variant of Uncertain Significance
ZSE: Zentrum für Seltene Erkrankungen, Center for Rare Diseases
